# Validation of High Ischemic and Bleeding Risk Criteria of European Guidelines in Peripheral Arterial Disease

**DOI:** 10.1016/j.jacasi.2025.01.018

**Published:** 2025-04-15

**Authors:** Kayo Yamamoto, Yuichi Saito, Yuji Ohno, Norikiyo Oka, Masayuki Takahara, Sakuramaru Suzuki, Raita Uchiyama, Masahiro Suzuki, Tadahiro Matsumoto, Yo Iwata, Yoshio Kobayashi

**Affiliations:** a Department of Cardiovascular Medicine, Chiba University Graduate School of Medicine, Chiba, Japan; b Department of Cardiology, Japanese Red Cross Narita Hospital, Narita, Japan; c Department of Cardiology, Funabashi Municipal Medical Center, Funabashi, Japan; d Department of Cardiology, Kimitsu Central Hospital, Kisarazu, Japan; e Department of Cardiology, Japan Community Health Organization Chiba Hospital, Chiba, Japan; f Department of Cardiology, Chibaken Saiseikai Narashino Hospital, Narashino, Japan

**Keywords:** guidelines, peripheral arterial disease, risk stratification

## Abstract

**Background:**

The 2024 European Society of Cardiology (ESC) guidelines for peripheral arterial disease (PAD) propose the dedicated high ischemic risk (HIR) and high bleeding risk (HBR) criteria.

**Objectives:**

The purpose of this study was to validate the ESC-HIR and HBR criteria using real-world data.

**Methods:**

From January 2019 to December 2022, this multicenter retrospective registry included 824 patients undergoing endovascular treatment for aortoiliac and femoropopliteal PAD. The ESC-HIR criteria include previous amputation, critical limb-threatening ischemia, previous revascularization, high-risk comorbidities (heart failure, diabetes, polyvascular disease), and estimated glomerular filtration rate <60 mL/min/1.73 m^2^, while the ESC-HBR criteria include dialysis or renal impairment (estimated glomerular filtration rate <15 mL/min/1.73 m^2^), acute coronary syndrome <30 days, history of stroke or transient ischemic attack, and active or clinically significant bleeding. Although patients were initially divided into 4 groups according to the presence or absence of HIR and HBR, patients with HBR and no HIR were excluded caused by the small sample size (n = 2). Major adverse cardiovascular and limb events and bleedings were evaluated.

**Results:**

Of the 822 patients, 62 (7.5%), 467 (56.8%), and 293 (35.6%) were grouped in the HIR (−)/HBR (−), HIR (+)/HBR (−), and HIR (+)/HBR (+). During the median follow-up period of 726 days, major adverse cardiovascular and limb events occurred in 0%, 9.5%, and 16.4% among the 3 groups *(P =* 0.005). The incidence of major bleeding events was 4.8%, 2.4%, and 6.8%, respectively *(P =* 0.009).

**Conclusions:**

The ESC-HIR and HBR criteria successfully stratified ischemic and bleeding risks in patients with PAD undergoing endovascular treatment.

Peripheral arterial disease (PAD) is a prevalent condition linked to systemic atherosclerosis, affecting over 200 million individuals globally with an increasing trend.^1^^,^^2^ Patients with PAD are at high ischemic risk (HIR) of serious cardiovascular and limb-related complications, such as myocardial infarction, stroke, acute limb ischemia, major amputations, and mortality.^3^, ^4^, ^5^ On the other hand, the presence of PAD is one of the factors associated with high bleeding risk (HBR).^6^ Despite recent advances in pharmacological and invasive therapeutic strategies, ischemic events and bleeding complications remain common, highlighting the need for carefully balanced antithrombotic treatment in patients with PAD.^4^ In 2024, for the ischemic and bleeding risk stratification, the European Society of Cardiology (ESC) proposed HIR and HBR criteria for patients with PAD, including medical history, comorbidities, and clinical presentations, aiding in prognostication and decision-making in tailored antithrombotic regimens.^7^ However, the ESC-HIR and HBR criteria have not been validated yet. This study aimed to evaluate the prognostic impact of the criteria on ischemic and bleeding outcomes in patients with PAD in a real-world setting.

## Methods

### Study population

**Figure 1:**
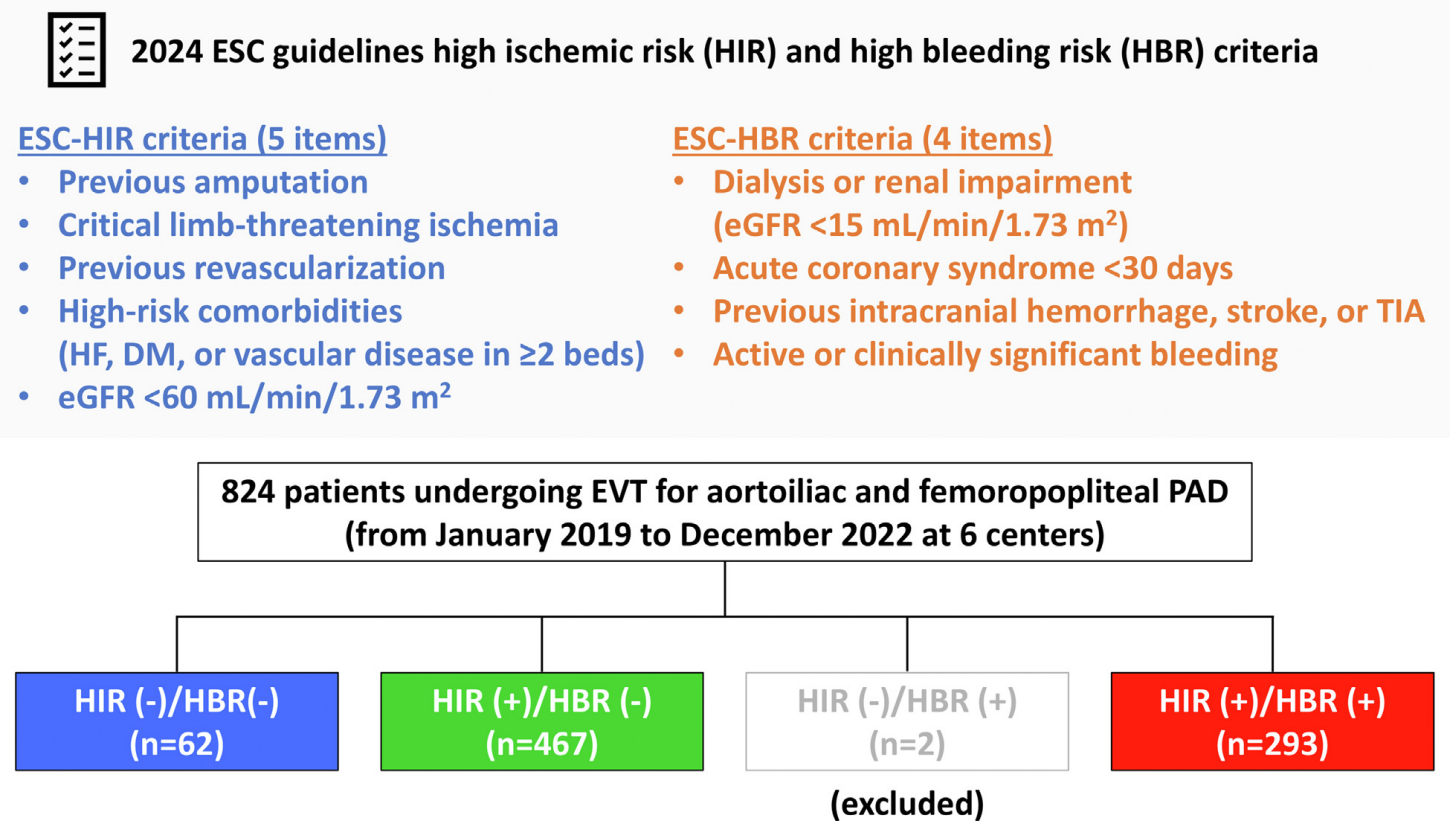
2024 ESC Guidelines HIR and HBR Criteria and Study Flow The European Society of Cardiology (ESC) proposed high ischemic risk (HIR) and high bleeding risk (HBR) criteria in 2024 for the ischemic and bleeding risk stratification in patients with peripheral arterial disease (PAD). According to the risk criteria, patients with PAD were divided into 4 groups. Patients with HBR and no HIR were excluded caused by the limited sample size. DM = diabetes; eGFR = estimated glomerular filtration rate; EVT = endovascular treatment; HF = heart failure; TIA = transient ischemic attack.

This was a multicenter, retrospective registry study. Between January 2019 and December 2022, a total of 824 patients with lower extremity PAD underwent endovascular treatment (EVT) for aortoiliac and femoropopliteal lesions at 6 hospitals in Japan (Funabashi Municipal Medical Center, Kimitsu Central Hospital, Japan Community Healthcare Organization Chiba Hospital, Chiba University Hospital, Narita Red Cross Hospital, and Chibaken Saiseikai Narashino Hospital). PAD was angiographically defined by occlusion or stenosis of the lower extremity arteries, regardless of ankle-brachial index. EVT was indicated for symptomatic PAD (Rutherford category 1-3) or chronic limb-threatening ischemia (CLTI) (Rutherford category 4-6).^7^ All EVT procedures were performed per local standard practice, with a bidirectional approach, intravascular imaging, drug-eluting stents, drug-coated balloons, and hemostasis devices.^8^, ^9^, ^10^, ^11^ Patients undergoing only below-the-knee artery revascularization were not included in the present study, but simultaneous EVT procedures for the below-the-knee arteries were allowed. Patients were initially divided into 4 groups according to the presence or absence of ESC-HIR and HBR but given the small sample size of those with HIR (−)/HBR (+), the patients (n = 2) were excluded (Figure 1). Thus, 822 patients with lower extremity PAD undergoing EVT were included in the present study. All participants provided written informed consent for the EVT procedures and informed consent for this study was ascertained in an opt-out manner. This study was done in accordance with the Declaration of Helsinki and centrally approved by the ethics committee of Chiba University Hospital.

### High ischemic and bleeding risk criteria

The criteria for ESC-HIR include previous amputation, CLTI, previous peripheral revascularization, high-risk comorbidities (ie, heart failure, diabetes, and vascular disease in ≥2 vascular beds), and estimated glomerular filtration rate <60 mL/min/1.73 m^2^, and the ESC-HBR criteria consist of dialysis or renal impairment (ie, estimated glomerular filtration rate <15 mL/min/1.73 m^2^), acute coronary syndrome within 30 days, history of intracranial hemorrhage, stroke or transient ischemic attack, and active or clinically significant bleeding (Figure 1).^7^ In the present study, a criterion of vascular disease in ≥2 vascular beds was considered positive when a history of coronary artery disease (CAD) or stroke was present in addition to PAD. In addition, a criterion of active or clinically significant bleeding was determined by a history of gastrointestinal bleeding events. Patients were defined as having HIR or HBR based on the presence of HIR or HBR criteria and were divided into 3 groups; HIR (−)/HBR (−), HIR (+)/HBR (−), and HIR (+)/HBR (+).

### Outcomes and statistical analysis

Clinical variables at baseline and outcomes after EVT were obtained from the medical records at the 6 hospitals. The coprimary outcomes of the present study included major adverse cardiovascular and limb events (MACLE) and major bleeding events. MACLE, a composite of cardiovascular death, myocardial infarction, ischemic stroke, acute limb ischemia, and major amputation,^12^ was defined according to the consensus documents,^13^, ^14^, ^15^ and major bleeding events were defined as Bleeding Academic Research Consortium type 3 or 5.^16^ The main interest of this study was to evaluate the relationships of ESC-HIR and HBR to ischemic and bleeding outcomes. Furthermore, the relationship between the number of ESC-HIR or HBR criteria and clinical outcomes was also assessed.

Statistical analysis was performed using JMP Pro 17.2.0 (SAS Institute). All data are expressed as mean ± SD, median (IQR), or frequency (percentage). Continuous variables were compared with analysis of variance or the Kruskal-Wallis test, while categorical variables were assessed using Fisher exact test. The Kaplan-Meier analysis was used to calculate the time to MACLE and major bleeding events after EVT, and the log-rank test was applied for between-group comparisons. The stratified analyses based on age, sex, and body mass index were also performed. The receiver-operating characteristic (ROC) curve analysis was performed to assess the diagnostic ability of the number ESC-HIR and HBR criteria for MACLE and major bleeding events with area under the curve (AUC). The best cutoff value was established by finding the values that corresponded to the maximum average sensitivity and specificity. A *P* value < 0.05 was considered statistically significant.

## Results

Of the 822 patients with lower extremity PAD undergoing EVT, 62 (7.5%), 467 (56.8%), and 293 (35.6%) were grouped in the HIR (−)/HBR (−), HIR (+)/HBR (−), and HIR (+)/HBR (+) (Figure 1). Baseline characteristics among the 3 groups are listed in Table 1. Overall, patients in the HIR (+)/HBR (+) group were likely to be complicated by more comorbidities, including atrial fibrillation, anemia, and renal dysfunction, than those in other groups. Patients in the HIR (+)/HBR (+) group were presented with CLTI in 62.1% and had polyvascular disease (a history of CAD and/or stroke plus PAD) in 78.5% and previous cerebrovascular events in 46.1% (Table 1). Procedural characteristics of EVT are shown in Table 2.

**Table 1:** Baseline Characteristics

	All (N = 822)	HIR (−)/ HBR (−) (n = 62)	HIR (+)/ HBR (−) (n = 467)	HIR (+)/ HBR (+) (n = 293)	*P* Value
Age, y	74.5 ± 8.8	73.2 ± 8.0	75.3 ± 8.6	73.4 ± 9.1	0.006
Men	605 (73.6)	48 (77.4)	326 (69.9)	231 (78.8)	0.02
Body mass index, kg/m^2^	22.5 ± 3.6	21.4 ± 2.8	22.7 ± 3.5	22.4 ± 3.8	0.02
Hypertension	670 (81.5)	49 (79.0)	372 (79.7)	249 (85.0)	0.15
Dyslipidemia	536 (65.2)	32 (51.6)	321 (68.7)	183 (62.4)	0.01
Current or previous smoking	519 (63.1)	51 (82.3)	315 (67.5)	153 (52.2)	<0.001
Previous CAD	314 (38.2)	0 (0)	171 (36.6)	143 (48.8)	<0.001
Atrial fibrillation	131 (15.9)	3 (4.8)	70 (15.0)	58 (19.8)	0.007
Hemoglobin, g/dL	12.4 ± 2.0	13.6 ± 1.6	12.7 ± 1.9	11.7 ± 1.9	<0.001
eGFR, mL/min/1.73 m^2^	50.5 (27.7-69.4)	76.9 (65.0-83.8)	55.7 (41.6-71.1)	10.3 (6.1-50.6)	<0.001
HbA1c, %	6.6 ± 1.2	5.8 ± 0.4	6.7 ± 1.2	6.6 ± 1.3	<0.001
LDL-C, mg/dL	94.2 ± 32.2	109.9 ± 32.3	95.9 ± 31.4	87.9 ± 32.1	<0.001
ESC-HIR criteria					
Previous amputation	5 (0.6)	0 (0)	3 (0.6)	2 (0.7)	1.00
CLTI	352 (42.8)	0 (0)	170 (36.4)	182 (62.1)	<0.001
Previous revascularization	179 (21.8)	0 (0)	104 (22.2)	75 (25.6)	<0.001
High-risk comorbidities	642 (78.1)	0 (0)	363 (77.7)	279 (95.2)	<0.001
Heart failure	125 (15.2)	0 (0)	64 (13.7)	61 (20.8)	<0.001
Diabetes	509 (61.9)	0 (0)	291 (62.3)	218 (74.4)	<0.001
Vascular disease in ≥2 beds	401 (48.8)	0 (0)	171 (36.6)	230 (78.5)	<0.001
eGFR <60 mL/min/1.73 m^2^	512 (62.3)	0 (0)	279 (59.7)	233 (79.5)	<0.001
ESC-HBR criteria					
Renal impairment	169 (20.6)	0 (0)	0 (0)	169 (57.7)	<0.001
ACS within 30 days	0 (0)	0 (0)	0 (0)	0 (0)	NA
Previous stroke or TIA	135 (16.4)	0 (0)	0 (0)	135 (46.1)	<0.001
Significant bleeding	30 (3.7)	0 (0)	0 (0)	30 (10.2)	<0.001
Medication					
Antithrombotic drugs	810 (98.5)	62 (100)	460 (98.5)	288 (98.3)	0.91
Aspirin	635 (77.5)	50 (80.6)	348 (74.7)	237 (81.4)	0.08
P2Y12 inhibitors	676 (82.2)	55 (88.7)	375 (80.3)	246 (84.0)	0.18
Cilostazol	193 (23.5)	17 (27.4)	130 (27.9)	46 (15.7)	<0.001
Oral anticoagulation	162 (19.7)	5 (8.1)	101 (21.6)	56 (19.1)	0.03
Low-dose rivaroxaban	2 (0.2)	0 (0)	2 (0.4)	0 ()	0.59
Statin	553 (67.4)	43 (69.4)	330 (71.0)	180 (61.4)	0.02
PPI	668 (83.7)	50 (80.7)	383 (82.0)	225 (87.0)	0.14
NSAIDs/steroids	106 (13.0)	7 (11.3)	61 (13.1)	38 (13.0)	0.97

Variables are mean ± SD, n (%), and median (IQR), or CLTI is defined as Rutherford category 4, 5, or 6. Renal impairment is defined as dialysis or eGFR <15 mL/min/1.73 m^2^. Significant bleeding is defined as active or history of clinically relevant gastrointestinal bleeding events.

ACS = acute coronary syndrome; CAD = coronary artery disease; CLTI = chronic limb-threatening ischemia; ESC = European Society of Cardiology; HBR = high bleeding risk; HIR = high ischemic risk; TIA = transient ischemic attack; eGFR = estimated glomerular filtration rate; HbA1c = hemoglobin A1c; LDL-C = low-density lipoprotein cholesterol; NA = not applicable; NSAID = non-steroidal anti-inflammatory drug; PPI = proton pump inhibitor; TIA = transient ischemic attack.

**Table 2:** Procedural Characteristics

	All (N = 822)	HIR (−)/ HBR (−) (n = 62)	HIR (+)/ HBR (−) (n = 467)	HIR (+)/ HBR (+) (n = 293)	*P* Value
Target lesion					
AI lesions only	212 (25.8)	29 (46.8)	124 (26.6)	59 (20.1)	<0.001
FP lesions only	361 (43.9)	24 (38.7)	213 (45.6)	124 (42.3)	0.48
Both AI and FP lesions	87 (10.6)	7 (11.3)	46 (9.9)	34 (11.6)	0.68
AI/FP and BTK lesions	162 (19.7)	2 (3.2)	84 (18.0)	76 (25.9)	<0.001
CTO	340 (41.4)	32 (51.6)	200 (42.8)	108 (36.9)	0.06
IVUS	654 (79.6)	54 (87.1)	403 (86.3)	197 (67.2)	<0.001
Devices used					
Bare metal stent	341 (41.5)	38 (61.3)	204 (43.7)	99 (33.8)	<0.001
Drug-coated balloon	299 (36.4)	19 (30.7)	168 (36.0)	112 (38.2)	0.52
Drug-eluting stent	226 (27.5)	12 (19.4)	128 (27.4)	86 (29.4)	0.28
Stent graft	39 (4.7)	1 (1.6)	25 (5.3)	13 (4.4)	0.48

Values are n (%).

AI = aortoiliac; BTK = below-the-knee; CTO = chronic total occlusion; FP = femoropopliteal; HBR = high bleeding risk; HIR = high ischemic risk; IVUS = intravascular ultrasound.

**Figure 2:**
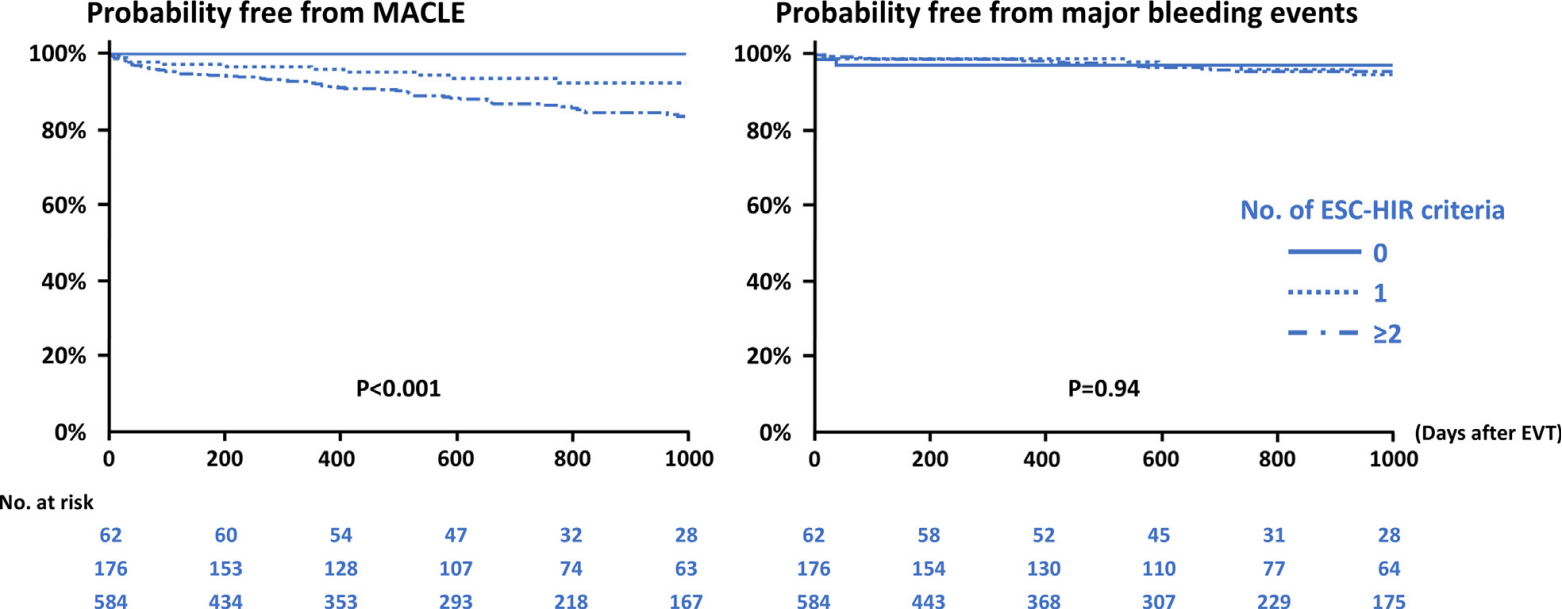
Clinical Outcomes According to the Number of HIR Criteria Ischemic and bleeding outcomes after EVT were evaluated. The number of HIR criteria was progressively associated with an increased major adverse cardiovascular and limb events (MACLE) risk, while it was not significantly related to the risk of major bleeding events. Abbeviations as in Figure 1.

**Figure 3:**
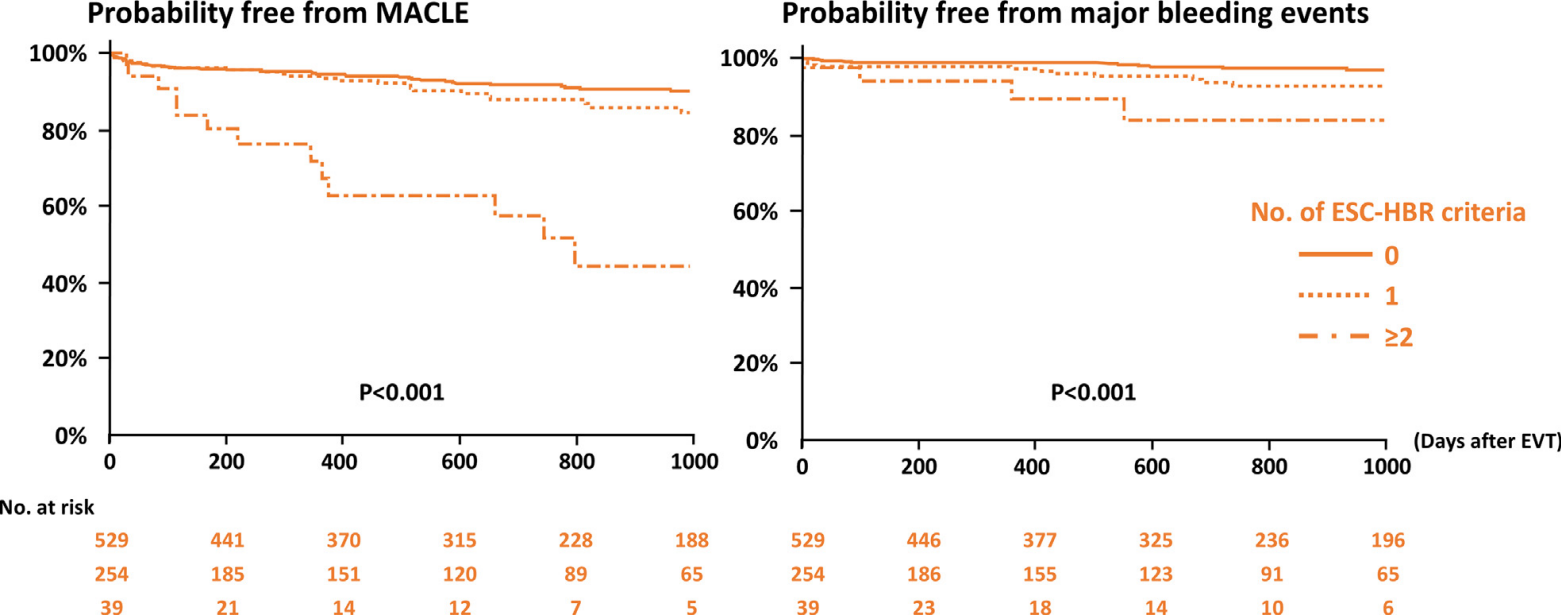
Clinical Outcomes According to the Number of HBR Criteria Ischemic and bleeding outcomes after EVT were evaluated. The number of HBR criteria was significantly associated with increased risks of MACLE and major bleeding events in a stepwise manner. Abbreviations as in Figures 1 and 2.

**Figure 4:**
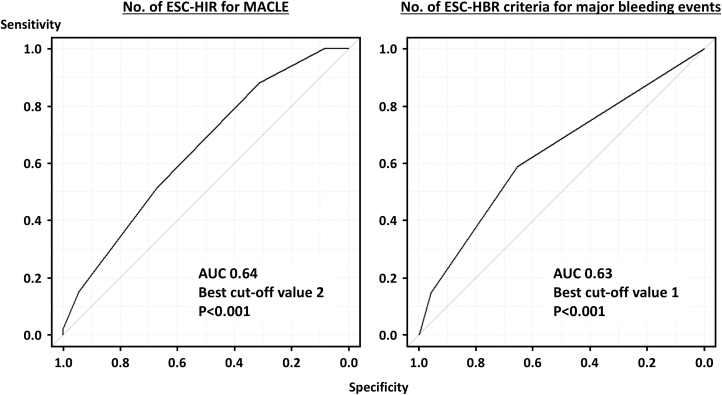
Receiver-Operating Characteristic Curve Analysis for MACLE and Major Bleedings The receiver-operating characteristic curve analysis showed that the number of ESC-HIR predicted MACLE with moderate diagnostic ability. Similarly, the number of ESC-HBR criteria were predictive of major bleeding events after endovascular treatment. AUC = area under the curve; abbreviations as in Figures 1 and 2.

**Figure 5:**
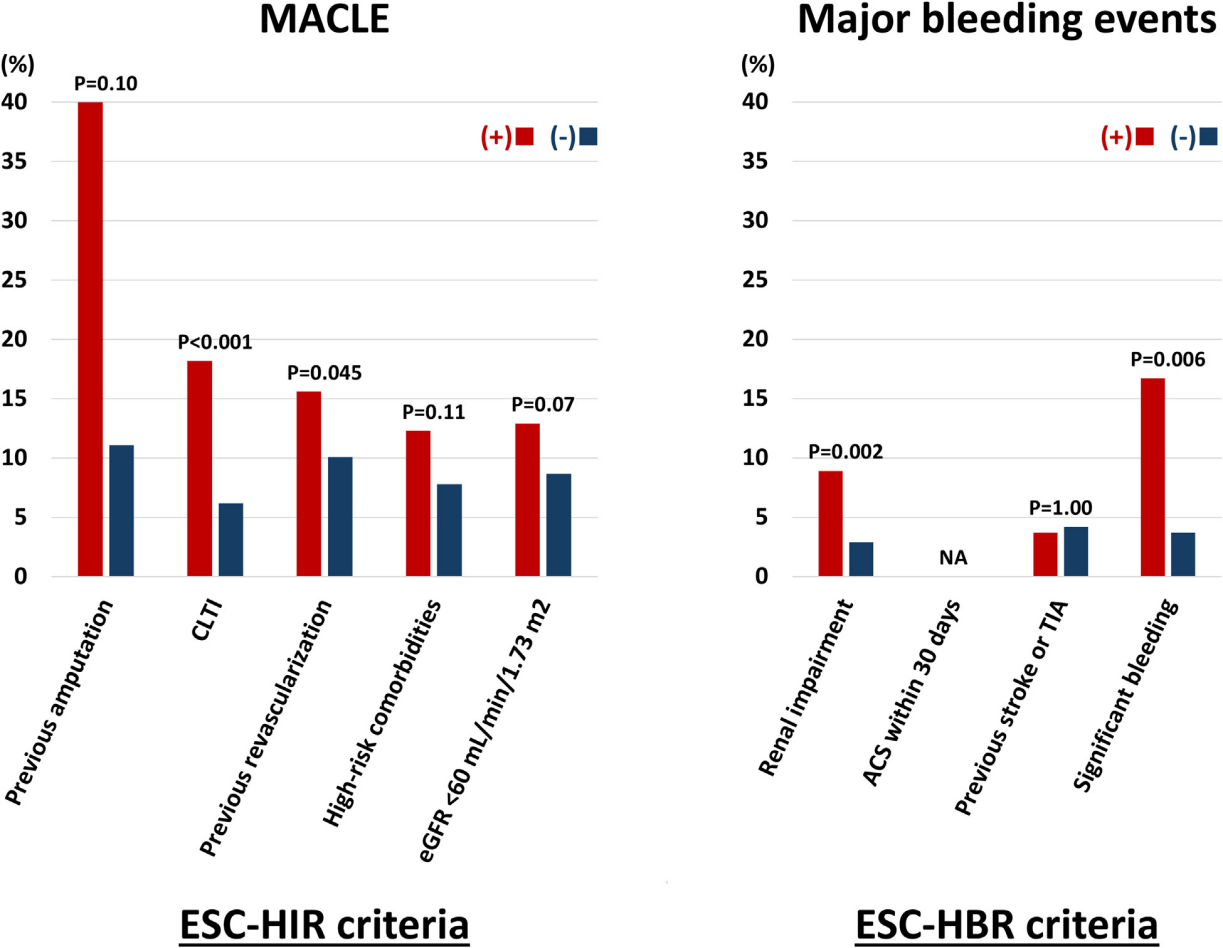
Prognostic Impact of Each Criterion of the ESC-HIR and HBR The rates of MACLE and major bleeding events were stratified by the individual ESC-HIR and HBR criteria. For ischemic risk stratification, the presence of CLTI and a history of previous revascularization were significantly associated with MACLE. For major bleeding events, renal impairment and active or clinically significant bleeding were particularly predictive. ACS = acute coronary syndrome; CLTI = critical limb-threatening ischemia; NA = not applicable; other abbreviations as in Figures 1 and 2.

During the median follow-up period of 726 days, 93 (11.3%) and 34 (4.1%) patients experienced MACLE and major bleeding events after EVT (Table 3). The Kaplan-Meier analysis demonstrated that the HIR (+)/HBR (+) group exhibited an increased risk of MACLE, followed by the HIR (+)/HBR (−) and HIR (−)/HBR (−) groups (Central Illustration). Patients in the HIR (+)/HBR (+) group also had a higher bleeding risk (Central Illustration). When patients were divided into 3 groups based on the number of ESC-HIR criteria (Supplemental Table 1)**,** those with 2 or more HIR criteria had an increased risk of MACLE compared with those with 0 or 1 criterion. On the other hand, there was no significant difference in the risk of major bleeding events according to the number of ESC-HIR criteria (Supplemental Table 2, Figure 2). In terms of ESC-HBR criteria, the greater number of criteria was significantly associated with higher risks of ischemic and bleeding outcomes in a stepwise manner (Supplemental Tables 3 and 4, Figure 3). The stratified analyses based on age, sex, and body mass index are displayed in Supplemental Figures 1 to 3, showing consistent results across the subgroups overall. The ROC curve analysis showed that the number of ESC-HIR (AUC: 0.64, best cutoff value 2; *P* < 0.001) and HBR (AUC: 0.63, best cutoff value 1; *P* < 0.001) criteria were predictive of MACLE and major bleeding events (Figure 4). With the best cutoff values, sensitivity, specificity, positive predictive value, negative predictive value, and accuracy were 88%, 31%, 14%, 95%, and 38% in the ESC-HIR criteria and 59%, 65%, 7%, 97%, and 65% in the ESC-HBR criteria, respectively. The rates of MACLE and major bleeding events were stratified by the individual ESC-HIR and HBR criteria in Figure 5. The presence of CLTI and previous revascularization were identified as significant factors associated with MACLE, while renal impairment and active or clinically significant bleeding were significantly related to major bleeding events (Figure 5).

**Table 3:** Clinical Outcomes

	All (N = 822)	HIR (−)/ HBR (−) (n = 62)	HIR (+)/ HBR (−) (n = 467)	HIR (+)/ HBR (+) (n = 293)	*P* Value
Follow-up, d	726 (318-1,131)	869 (612-1,359)	739 (366-1,218)	576 (170-1,007)	<0.001
MACLE	93 (11.3)	0 (0)	45 (9.5)	48 (16.4)	<0.001
Cardiovascular death	31 (3.8)	0 (0)	10 (2.1)	21 (7.2)	<0.001
Myocardial infarction	11 (1.3)	0 (0)	7 (1.5)	4 (1.4)	1.00
Ischemic stroke	25 (3.0)	0 (0)	14 (3.0)	11 (3.8)	0.34
Acute limb ischemia	9 (1.1)	0 (0)	4 (0.9)	5 (1.7)	0.40
Major amputation	32 (3.9)	0 (0)	13 (2.8)	19 (6.5)	0.01
Major bleeding events	34 (4.1)	3 (4.8)	11 (2.4)	20 (6.8)	0.009
All-cause death	156 (19.0)	6 (9.7)	71 (15.2)	79 (27.0)	<0.001

Values are median (IQR) or n (%).

MACLE = major adverse cardiovascular and limb events; other abbreviations as in Table 1.

**Central Illustration:**
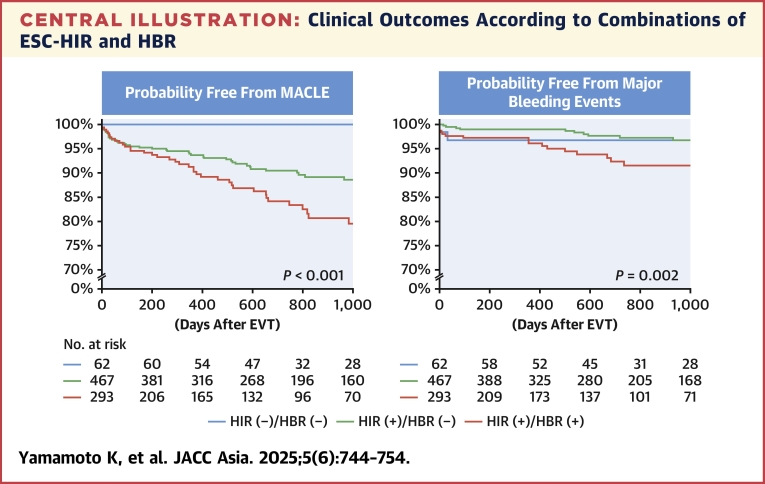
Clinical Outcomes According to Combinations of ESC-HIR and HBR Combinations of European Society of Cardiology (ESC) high ischemic risk (HIR) and high bleeding risk (HBR) criteria were significantly associated with major adverse cardiovascular and limb events (MACLE) and major bleeding events after endovascular treatment (EVT) in patients with peripheral arterial disease. Patients without HIR had a lower ischemic event risk than those with at least 1 ESC-HIR criteria. The risk of MACLE in patients with HIR was further stratified according to the ESC-HBR criteria. Patients with both HIR and HBR had a higher bleeding risk.

## Discussion

This multicenter registry study aimed to validate the HIR and HBR criteria proposed in the ESC guidelines 2024, showcasing the successful risk stratification in patients with lower extremity PAD undergoing EVT for aortoiliac and femoropopliteal lesions. When an HBR criterion was present, almost all (293 of 295, 99.3%) patients had concomitant HIR. In patients without HIR, none experienced MACLE during the follow-up period in the present study, while those with HIR had an increased risk of ischemic outcomes, particularly when complicated by HBR. Patients having HBR were more likely to develop major bleeding events than their counterparts. In addition, the increase in the number of HIR and HBR criteria was progressively associated with higher ischemic and bleeding risks. Our results suggest that the ESC-HIR and HBR may be useful for decision-making in tailored management including antithrombotic regimens in patients with PAD.

### Ischemic and bleeding risks in PAD

It is well known that patients with PAD are at HIR and HBR. A large-scale, single-center, prospective study in the Netherlands showed that patients with PAD more frequently experienced major adverse cardiovascular and, as expected, limb events than those with coronary and cerebrovascular diseases.^5^ Additionally, the pivotal COMPASS (Cardiovascular Outcomes for People Using Anticoagulation Strategies) randomized trial, in which patients with CAD, PAD, or both were included to test the hypothesis if low-dose rivaroxaban (2.5 mg twice daily) on top of aspirin or rivaroxaban alone is beneficial to reduce ischemic events, also demonstrated that patients with PAD had a higher risk of cardiovascular death, stroke, or myocardial infarction than those without (6.0% vs 4.3%) during a mean follow-up period of 23 months.^17^ Given the high ischemic event rate, it is conceivable that potent antithrombotic therapies may be beneficial in patients with PAD. Indeed, the COMPASS and subsequent VOYAGER PAD (Vascular Outcomes Study of ASA [acetylsalicylic acid] Along with Rivaroxaban in Endovascular or Surgical Limb Revascularization for PAD [peripheral artery disease]) trials have established the effectiveness of low-dose rivaroxaban plus aspirin in reducing MACLE in high-risk PAD.^12^^,^^17^ On the other hand, domestic guidelines in Japan for antithrombotic therapy in patients with CAD indicate that the presence of PAD is a strong predictor of bleeding outcomes.^6^ Therefore, accurate risk stratification is clinically relevant in this patient population of HIR and HBR. In the field of CAD, the Academic Research Consortium proposed criteria for defining HBR in patients undergoing percutaneous coronary intervention, which is currently widely used in daily practice.^18^ In the field of PAD, however, no universal definitions to determine HIR and HBR have been established. Although the HBR criteria proposed by the Academic Research Consortium reportedly stratified ischemic and bleeding risks in PAD as well as CAD,^19^ a dedicated risk-predicting system has been warranted. In this context, the 2024 ESC guidelines for PAD proposed the criteria for HIR and HBR in this specific patient population.^7^ The guidelines also recommend tailoring antithrombotic regimens using the HIR and HBR criteria in patients with PAD. For instance, in patients with chronic symptomatic PAD and no HIR, single antiplatelet therapy (aspirin or clopidogrel) is recommended (Class I), while aspirin plus low-dose rivaroxaban should be considered in those with HIR and no HBR (Class IIa).^7^ However, before the widespread dissemination of the ESC-HIR and HBR criteria to guide the management and antithrombotic regimens of patients with PAD in a real-world practice, external validation is needed. In general, (East) Asian populations have a relatively lower ischemic risk and higher bleeding risk as compared to the Western counterparts in the field of cardiovascular disease.^20^ In patients with PAD, there may also be a racial difference in clinical outcomes. A meta-analysis comparing Black and White races reported that Black patients had an increased risk of major amputation within 30 days, while White patients had a higher myocardial infarction risk after interventional procedures for PAD.^21^ Another previous study comparing White and Asian patients with PAD undergoing EVT across the United States, Canada, and Singapore indicated that Asian patients had worse in-hospital outcomes and long-term patency after the procedures.^22^ In this context, we validated the ESC-HIR and HBR criteria in a Japanese PAD population.

### Clinical implications

The ESC-HIR and HBR criteria were mainly driven by the inclusion and exclusion criteria of the COMPASS and VOYAGER PAD trials.^12^^,^^17^ In the present study, MACLE was defined with the same components in the VOYAGER PAD trial. Overall, the incidence of MACLE in the VOYAGER PAD was 17.3% and 19.9% at 3 years in the low-dose rivaroxaban and placebo groups, which is in line with our results (ie, 11.3% during the median of 726 days). One of the important findings in the present study may include the low event rate (0%) in the no-HIR group, suggesting that additional potent antithrombotic therapy may not be needed (Central Illustration). In contrast, patients with at least 1 HIR criteria had an increased risk of MACLE. Given that a bleeding risk was relatively low and similar between the groups of HIR (−)/HBR (−) and HIR (+)/HBR (−), the latter may be candidates for potent antithrombotic strategies including low-dose rivaroxaban, as recommended by the ESC guidelines.^7^ In a setting of CAD and PAD, patients with HBR usually have HIR and vice versa,^23^, ^24^, ^25^ making tailored management and antithrombotic regimens challenging. Interestingly, while the greater number of HBR criteria was progressively related to increased ischemic and bleeding risks, the number of HIR criteria was exclusively associated with ischemic rather than bleeding risks in the present study (Figure 2). Thus, ESC-HIR criteria may be particularly useful in stratifying ischemic event risk in patients with PAD. In particular, CLTI and previous limb revascularization were associated with ischemic events, and renal impairment and a history of bleeding were identified as predictors of major bleeding events after EVT. Given the high negative predictive value, the ESC-HIR and HBR criteria may help identify patients who are unlikely to develop ischemic and bleeding events. Although it remains uncertain whether antithrombotic strategies based on ESC-HIR and HBR improve clinical outcomes, we believe that the criteria have the potential to stratify ischemic and bleeding risks appropriately and aid in the management of this vulnerable patient population. For instance, in the HIR (−)/HBR (−) group, conventional single antiplatelet therapy with aspirin or clopidogrel may be reasonable because both ischemic and bleeding risks were low. In the HIR (+)/HBR (−) group, dual antithrombotic therapy with aspirin and low-dose rivaroxaban is a potential therapeutic option as indicated in the ESC guidelines.^7^ For patients in the HIR (+)/HBR (+) group, a tailored and balanced approach may be warranted. Further studies are needed to test the effectiveness of the ESC-HIR and HBR criteria as a guiding tool for optimizing antithrombotic regimens.

### Study limitations

This was a retrospective study with a moderate sample size, in which some criteria were slightly modified. For instance, an HBR criterion of active or clinically significant bleeding was determined by a history of gastrointestinal bleeding events in the present study, although it was defined as gastrointestinal ulceration, presence of malignant neoplasms at high risk of bleeding, current or recent brain or spinal injury, known esophageal varices, and vascular aneurysms of the large arteries or major intraspinal or intracerebral vascular abnormalities in the VOYAGER PAD trial.^12^ However, the gastrointestinal tract is the most common etiology of major bleeding events in patients with PAD as shown in a sub-analysis of the COMPASS trial.^26^ Because of the limited overall sample size and the number of events, the prognostic impact of HIR and HBR criteria on individual components of MACLE remains unclear. Similarly, the sample size prevented sub-analyses per EVT device used (ie, drug-coated balloons and drug-eluting stents) and the site-level analysis. In the present study, patients undergoing EVT for aortoiliac and femoropopliteal PAD were exclusively included. These inclusion criteria may be in line with those in the COMPASS and VOYAGER PAD trials,^12^^,^^17^ but the diagnostic ability of the ESC-HIR and HBR criteria outside of our study population (eg, patients with exclusive infrapopliteal artery lesions and non-Asian populations) is still uncertain. Furthermore, because few patients (0.2%) received low-dose rivaroxaban in this study, the impact of the antithrombotic regimen on ischemic and bleeding outcomes was unknown. In addition, details of MACLE and major bleeding events (eg, specific therapeutic strategies for events and bleeding sites) were lacking, and data on pharmacological treatment during the follow-up period were unavailable.

## Conclusions

The HIR and HBR criteria proposed in the ESC guidelines 2024 successfully stratified risks of cardiovascular and limb-related outcomes and major bleeding events in patients with lower-extremity PAD undergoing EVT. Our findings support the use of these criteria to guide clinical decision-making in individualized management and antithrombotic regimens in this vulnerable population.

## Funding Support and Author Disclosures

The authors have reported that they have no relationships relevant to the contents of this paper to disclose.

## References

[1] Fowkes F.G., Rudan D., Rudan I. (2013). Comparison of global estimates of prevalence and risk factors for peripheral artery disease in 2000 and 2010: a systematic review and analysis. Lancet.

[2] GBD 2019 Peripheral Artery Disease Collaborators (2023). Global burden of peripheral artery disease and its risk factors, 1990-2019: a systematic analysis for the Global Burden of Disease Study 2019. Lancet Glob Health.

[3] Alberts M.J., Bhatt D.L., Mas J.L. (2009). Three-year follow-up and event rates in the international REduction of Atherothrombosis for Continued Health Registry. Eur Heart J.

[4] Anand S.S., Caron F., Eikelboom J.W. (2018). Major adverse limb events and mortality in patients with peripheral artery disease: the COMPASS trial. J Am Coll Cardiol.

[5] Hageman S.H.J., de Borst G.J., Dorresteijn J.A.N. (2020). Cardiovascular risk factors and the risk of major adverse limb events in patients with symptomatic cardiovascular disease. Heart.

[6] Nakamura M., Kimura K., Kimura T. (2020). JCS 2020 guideline focused update on antithrombotic therapy in patients with coronary artery disease. Circ J.

[7] Mazzolai L., Teixido-Tura G., Lanzi S. (2024). 2024 ESC guidelines for the management of peripheral arterial and aortic diseases. Eur Heart J.

[8] Suzuki K., Ueshima D., Higashitani M. (2023). Two-year results of endovascular therapy for femoropopliteal artery disease in Japan during the introduction of drug-eluting devices. Cardiovasc Interv Ther.

[9] Takamatsu S., Kagiyama N., Sone N. (2023). Impact of radial compression protocols on radial artery occlusion and hemostasis time in coronary angiography. Cardiovasc Interv Ther.

[10] Yu A., Fujimura N., Hayashi M. (2024). Hybrid treatment of distal bypass and pedal artery angioplasty following intraoperative direct puncture angiography for chronic limb-threatening ischemia with occult vessel. Cardiovasc Interv Ther.

[11] Shima Y., Taninobu N., Ikuta A. (2024). Two-year outcomes of endovascular therapy for femoropopliteal arterial lesions for patients with high bleeding risk. Cardiovasc Interv Ther.

[12] Bonaca M.P., Bauersachs R.M., Anand S.S. (2020). Rivaroxaban in peripheral artery disease after revascularization. N Engl J Med.

[13] Hicks K.A., Mahaffey K.W., Mehran R. (2018). 2017 Cardiovascular and stroke endpoint definitions for clinical trials. Circulation.

[14] Lakhter V., Weinberg M.D., Galmer A. (2021). Objective outcome measures for trials in patients with chronic limb-threatening ischemia across 2 decades: analysis and recommendations. JACC Cardiovasc Interv.

[15] Gornik H.L., Aronow H.D., Goodney P.P. (2024). 2024 ACC/AHA/AACVPR/APMA/ABC/SCAI/SVM/SVN/SVS/SIR/VESS guideline for the management of lower extremity peripheral artery disease: a report of the American College of Cardiology/American Heart Association Joint Committee on Clinical Practice Guidelines. J Am Coll Cardiol.

[16] Mehran R., Rao S.V., Bhatt D.L. (2011). Standardized bleeding definitions for cardiovascular clinical trials: a consensus report from the Bleeding Academic Research Consortium. Circulation.

[17] Eikelboom J.W., Connolly S.J., Bosch J. (2017). Rivaroxaban with or without aspirin in stable cardiovascular disease. N Engl J Med.

[18] Urban P., Mehran R., Colleran R. (2019). Defining high bleeding risk in patients undergoing percutaneous coronary intervention: a consensus document from the Academic Research Consortium for High Bleeding Risk. Eur Heart J.

[19] Tomoi Y., Kuramitsu S., Shinozaki T. (2023). Validation of the Academic Research Consortium High Bleeding Risk (ARC-HBR) criteria in patients undergoing peripheral endovascular interventions. EuroIntervention.

[20] Levine G.N., Jeong Y.H., Goto S. (2014). Expert consensus document: World Heart Federation expert consensus statement on antiplatelet therapy in East Asian patients with ACS or undergoing PCI. Nat Rev Cardiol.

[21] Jaiswal V., Hanif M., Ang S.P. (2023). Racial disparity between the post-procedural outcomes among patients with peripheral artery disease: a systematic review and meta-analysis. Curr Probl Cardiol.

[22] Chen P., Patel P.B., Ding J. (2023). Asian race is associated with peripheral arterial disease severity and postoperative outcomes. J Vasc Surg.

[23] Matsumoto T., Saito Y., Sato T. (2023). Validation of the domestic high bleeding risk criteria for japanese patients with acute myocardial infarction. J Atheroscler Thromb.

[24] Matsumoto T., Saito Y., Sato T. (2024). Diagnostic ability of Japanese version of high bleeding risk criteria for ischemic outcomes in patients with acute myocardial infarction. Heart Vessels.

[25] Nakanishi N., Kaikita K., Ishii M. (2023). Japanese high bleeding risk criteria status predicts low thrombogenicity and bleeding events in patients undergoing percutaneous coronary intervention. Cardiovasc Interv Ther.

[26] Eikelboom J.W., Bosch J.J., Connolly S.J. (2019). Major bleeding in patients with coronary or peripheral artery disease treated with rivaroxaban plus aspirin. J Am Coll Cardiol.

