# Supplementary material for: Validation of High Ischemic and Bleeding Risk Criteria of European Guidelines in Peripheral Arterial Disease
Source: JACC Asia. 2025 Apr 15;5(6):744–54. doi: 10.1016/j.jacasi.2025.01.018 (PMC12287741; doi:10.1016/j.jacasi.2025.01.018)
Supplement: Supplementary Tables 1-4 and Supplementary Figure 1-3 [file mmc1.docx]

**Supplemental Table 1. Baseline characteristics**

| Variable | All  (n=822) | Number of ESC-HIR criteria | | | P value |
| --- | --- | --- | --- | --- | --- |
|  |  | 0  (n=62) | 1  (n=176) | ≥2  (n=584) |  |
| Age (years) | 74.5±8.8 | 73.2±8.0 | 72.7±8.6 | 75.1±8.9 | 0.03 |
| Men | 605 (73.6%) | 48 (77.4%) | 135 (76.7%) | 422 (72.3%) | 0.41 |
| Body mass index (kg/m^2^) | 22.5±3.6 | 21.4±2.8 | 23.0±3.9 | 22.5±3.5 | 0.01 |
| Hypertension | 670 (81.5%) | 49 (79.0%) | 140 (79.6%) | 481 (82.4%) | 0.57 |
| Dyslipidemia | 536 (65.2%) | 32 (51.6%) | 123 (69.9%) | 381 (65.2%) | 0.04 |
| Current or previous smoking | 519 (63.1%) | 51 (82.3%) | 126 (71.6%) | 342 (58.6%) | <0.001 |
| Previous CAD | 314 (38.2%) | 0 (0%) | 42 (23.9%) | 272 (46.6%) | <0.001 |
| Atrial fibrillation | 131 (15.9%) | 3 (4.8%) | 19 (10.8%) | 109 (18.7%) | 0.001 |
| Hemoglobin (g/dL) | 12.4±2.0 | 13.6±1.6 | 13.2±1.8 | 12.0±1.9 | <0.001 |
| eGFR (mL/min/1.73 m^2^) | 50.5 [27.7, 69.4] | 76.9 [65.0, 83.8] | 69.5 [60.8, 81.0] | 39.8 [11.6, 56.3] | <0.001 |
| HbA1c (%) | 6.6±1.2 | 5.8±0.4 | 6.4±1.0 | 6.7±1.3 | <0.001 |
| LDL-C (mg/dL) | 94.2±32.2 | 109.9±32.3 | 96.5±28.0 | 91.7±33.0 | <0.001 |
| ESC-HIR criteria |  |  |  |  |  |
| Previous amputation | 5 (0.6%) | 0 (0%) | 0 (0%) | 5 (0.9%) | 0.73 |
| CLTI | 352 (42.8%) | 0 (0%) | 21 (11.9%) | 331 (56.7%) | <0.001 |
| Previous revascularization | 179 (21.8%) | 0 (0%) | 9 (5.1%) | 170 (29.1%) | <0.001 |
| High-risk comorbidities | 642 (78.1%) | 0 (0%) | 105 (59.7%) | 537 (92.0%) | <0.001 |
| Heart failure | 125 (15.2%) | 0 (0%) | 8 (4.6%) | 117 (20.3%) | <0.001 |
| Diabetes | 509 (61.9%) | 0 (0%) | 77 (43.6%) | 432 (74.0%) | <0.001 |
| Vascular disease in ≥2 beds | 401 (48.8%) | 0 (0%) | 56 (31.8%) | 345 (59.1%) | <0.001 |
| eGFR <60 mL/min/1.73 m^2^ | 512 (62.3%) | 0 (0%) | 41 (23.3%) | 471 (80.7%) | <0.001 |
| ESC-HBR criteria |  |  |  |  |  |
| Renal impairment | 169 (20.6%) | 0 (0%) | 4 (2.3%) | 165 (28.3%) | <0.001 |
| ACS within 30 days | 0 (0%) | 0 (0%) | 0 (0%) | 0 (0%) | NA |
| Previous stroke or TIA | 135 (16.4%) | 0 (0%) | 18 (10.2%) | 117 (20.0%) | <0.001 |
| Significant bleeding | 30 (3.7%) | 0 (0%) | 3 (1.7%) | 27 (4.6%) | 0.06 |
| Medication |  |  |  |  |  |
| Antithrombotic drugs | 810 (98.5%) | 62 (100%) | 175 (99.4%) | 573 (98.1%) | 0.46 |
| Aspirin | 635 (77.5%) | 50 (80.6%) | 141 (80.1%) | 444 (76.4%) | 0.52 |
| P2Y12 inhibitors | 676 (82.2%) | 55 (88.7%) | 151 (85.8%) | 470 (80.5%) | 0.11 |
| Cilostazol | 193 (23.5%) | 17 (27.4%) | 45 (25.6%) | 131 (22.5%) | 0.49 |
| Oral anticoagulation | 162 (19.7%) | 5 (8.1%) | 29 (16.5%) | 128 (21.9%) | 0.01 |
| Low-dose rivaroxaban | 2 (0.2%) | 0 (0%) | 1 (0.6%) | 1 (0.2%) | 0.50 |
| Statin | 553 (67.4%) | 43 (69.4%) | 128 (73.6%) | 382 (65.4%) | 0.13 |
| PPI | 668 (83.7%) | 50 (80.7%) | 141 (80.1%) | 497 (85.1%) | 0.22 |
| NSAIDs/steroids | 106 (13.0%) | 7 (11.3%) | 21 (11.9%) | 78 (13.4%) | 0.87 |

CLTI is defined as Rutherford category 4, 5, or 6. Renal impairment is defined as dialysis or eGFR <15 mL/min/1.73 m^2^. Significant bleeding is defined as active or history of clinically relevant gastrointestinal bleeding events.

ACS = acute coronary syndrome; CAD = coronary artery disease; CLTI = chronic limb-threatening ischemia; ESC = European Society of Cardiology; HBR = high bleeding risk; HIR = high ischemic risk; TIA = transient ischemic attack; eGFR = estimated glomerular filtration rate; HbA1c = hemoglobin A1c; LDL-C = low-density lipoprotein cholesterol; NA = not applicable; NSAIDs = non-steroidal anti-inflammatory drugs; PPI = proton pump inhibitor; TIA = transient ischemic attack.

**Supplemental Table 2. Clinical outcomes**

| Variable | All  (n=822) | Number of ESC-HIR criteria | | | P value |
| --- | --- | --- | --- | --- | --- |
|  |  | 0  (n=62) | 1  (n=176) | ≥2  (n=584) |  |
| Follow-up (days) | 726 [318, 1131] | 869 [612, 1359] | 738 [384, 1180] | 673 [243, 1108] | <0.001 |
| MACLE | 93 (11.3%) | 0 (0%) | 11 (6.3%) | 82 (14.0%) | <0.001 |
| Cardiovascular death | 31 (3.8%) | 0 (0%) | 0 (0%) | 31 (5.3%) | <0.001 |
| Myocardial infarction | 11 (1.3%) | 0 (0%) | 3 (1.7%) | 8 (1.4%) | 0.77 |
| Ischemic stroke | 25 (3.0%) | 0 (0%) | 3 (1.7%) | 22 (3.8%) | 0.16 |
| Acute limb ischemia | 9 (1.1%) | 0 (0%) | 0 (0%) | 9 (1.5%) | 0.28 |
| Major amputation | 32 (3.9%) | 0 (0%) | 5 (2.8%) | 27 (4.7%) | 0.15 |
| Major bleeding events | 34 (4.1%) | 3 (4.8%) | 7 (4.0%) | 24 (4.1%) | 0.91 |
| All-cause death | 156 (19.0%) | 6 (9.7%) | 16 (9.1%) | 134 (23.0%) | <0.001 |

HIR = high ischemic risk; MACLE = major adverse cardiovascular and limb events.

**Supplemental Table 3. Baseline characteristics**

| Variable | All  (n=822) | Number of ESC-HBR criteria | | | P value |
| --- | --- | --- | --- | --- | --- |
|  |  | 0  (n=529) | 1  (n=254) | ≥2  (n=39) |  |
| Age (years) | 74.5±8.8 | 75.1±8.6 | 73.3±9.3 | 73.8±8.2 | 0.03 |
| Men | 605 (73.6%) | 374 (70.7%) | 203 (80.0%) | 28 (71.8%) | 0.02 |
| Body mass index (kg/m^2^) | 22.5±3.6 | 22.6±3.4 | 22.5±3.8 | 21.8±4.2 | 0.44 |
| Hypertension | 670 (81.5%) | 421 (79.6%) | 218 (85.8%) | 31 (79.5%) | 0.09 |
| Dyslipidemia | 536 (65.2%) | 353 (66.7%) | 162 (63.8%) | 21 (53.9%) | 0.22 |
| Current or previous smoking | 519 (63.1%) | 366 (69.2%) | 136 (53.5%) | 17 (43.6%) | <0.001 |
| Previous CAD | 314 (38.2%) | 171 (32.3%) | 121 (47.6%) | 22 (56.4%) | <0.001 |
| Atrial fibrillation | 131 (15.9%) | 73 (13.8%) | 46 (18.1%) | 12 (30.8%) | 0.01 |
| Hemoglobin (g/dL) | 12.4±2.0 | 12.8±1.9 | 11.8±1.9 | 10.8±1.7 | <0.001 |
| eGFR (mL/min/1.73 m^2^) | 50.5 [27.7, 69.4] | 58.8 [43.3, 75.0] | 12.6 [6.1, 54.9] | 7.1 [5.9, 12.5] | <0.001 |
| HbA1c (%) | 6.6±1.2 | 6.6±1.2 | 6.6±1.2 | 6.5±1.7 | 0.80 |
| LDL-C (mg/dL) | 94.2±32.2 | 97.5±31.8 | 89.0±32.4 | 80.1±29.7 | <0.001 |
| ESC-HIR criteria |  |  |  |  |  |
| Previous amputation | 5 (0.6%) | 3 (0.6%) | 2 (0.8%) | 0 (0%) | 0.73 |
| CLTI | 352 (42.8%) | 170 (32.1%) | 152 (59.8%) | 30 (76.9%) | <0.001 |
| Previous revascularization | 179 (21.8%) | 104 (19.7%) | 60 (23.6%) | 15 (38.5%) | 0.02 |
| High-risk comorbidities | 642 (78.1%) | 363 (68.6%) | 240 (94.5%) | 39 (100%) | <0.001 |
| Heart failure | 125 (15.2%) | 64 (12.1%) | 50 (19.7%) | 11 (28.2%) | 0.002 |
| Diabetes | 509 (61.9%) | 291 (55.0%) | 186 (73.2%) | 32 (82.1%) | <0.001 |
| Vascular disease in ≥2 beds | 401 (48.8%) | 171 (32.3%) | 193 (76.0%) | 37 (94.9%) | <0.001 |
| eGFR <60 mL/min/1.73 m^2^ | 512 (62.3%) | 279 (52.7%) | 196 (77.2%) | 37 (94.9%) | <0.001 |
| ESC-HBR criteria |  |  |  |  |  |
| Renal impairment | 169 (20.6%) | 0 (0%) | 136 (53.5%) | 33 (84.6%) | <0.001 |
| ACS within 30 days | 0 (0%) | 0 (0%) | 0 (0%) | 0 (0%) | NA |
| Previous stroke or TIA | 135 (16.4%) | 0 (0%) | 101 (39.8%) | 34 (87.2%) | <0.001 |
| Significant bleeding | 30 (3.7%) | 0 (0%) | 17 (6.7%) | 13 (33.3%) | <0.001 |
| Medication |  |  |  |  |  |
| Antithrombotic drugs | 810 (98.5%) | 522 (98.7%) | 250 (98.4%) | 38 (97.4%) | 0.56 |
| Aspirin | 635 (77.5%) | 398 (75.4%) | 205 (81.0%) | 32 (84.2%) | 0.13 |
| P2Y12 inhibitors | 676 (82.2%) | 430 (81.3%) | 214 (84.3%) | 32 (82.1%) | 0.60 |
| Cilostazol | 193 (23.5%) | 147 (27.8%) | 41 (16.1%) | 5 (12.8%) | <0.001 |
| Oral anticoagulation | 162 (19.7%) | 106 (20.0%) | 48 (18.9%) | 8 (20.5%) | 0.92 |
| Low-dose rivaroxaban | 2 (0.2%) | 2 (0.4%) | 0 (0%) | 0 (0%) | 1.00 |
| Statin | 553 (67.4%) | 373 (70.8%) | 158 (62.2%) | 22 (56.4%) | 0.02 |
| PPI | 668 (83.7%) | 433 (81.9%) | 221 (87.0%) | 34 (87.2%) | 0.17 |
| NSAIDs/steroids | 106 (13.0%) | 68 (12.9%) | 28 (11.0%) | 10 (25.6%) | 0.052 |

CLTI is defined as Rutherford category 4, 5, or 6. Renal impairment is defined as dialysis or eGFR <15 mL/min/1.73 m^2^. Significant bleeding is defined as active or history of clinically relevant gastrointestinal bleeding events.

ACS = acute coronary syndrome; CAD = coronary artery disease; CLTI = chronic limb-threatening ischemia; ESC = European Society of Cardiology; HBR = high bleeding risk; HIR = high ischemic risk; TIA = transient ischemic attack; eGFR = estimated glomerular filtration rate; HbA1c = hemoglobin A1c; LDL-C = low-density lipoprotein cholesterol; NA = not applicable; NSAIDs = non-steroidal anti-inflammatory drugs; PPI = proton pump inhibitor; TIA = transient ischemic attack.

**Supplemental Table 4. Clinical outcomes**

| Variable | All  (n=822) | Number of ESC-HBR criteria | | | P value |
| --- | --- | --- | --- | --- | --- |
|  |  | 0  (n=529) | 1  (n=254) | ≥2  (n=39) |  |
| Follow-up (days) | 726 [318, 1131] | 755 [371, 1220] | 599 [180, 1020] | 367 [93, 819] | <0.001 |
| MACLE | 93 (11.3%) | 45 (8.5%) | 33 (12.0%) | 15 (38.5%) | <0.001 |
| Cardiovascular death | 31 (3.8%) | 10 (1.9%) | 14 (5.5%) | 7 (18.0%) | <0.001 |
| Myocardial infarction | 11 (1.3%) | 7 (1.3%) | 3 (1.2%) | 1 (2.6%) | 0.52 |
| Ischemic stroke | 25 (3.0%) | 14 (2.7%) | 6 (2.4%) | 5 (12.8%) | 0.01 |
| Acute limb ischemia | 9 (1.1%) | 4 (0.8%) | 3 (1.2%) | 2 (5.1%) | 0.07 |
| Major amputation | 32 (3.9%) | 13 (2.5%) | 14 (5.5%) | 5 (12.8%) | 0.003 |
| Major bleeding events | 34 (4.1%) | 14 (2.7%) | 15 (5.9%) | 5 (12.8%) | 0.003 |
| All-cause death | 156 (19.0%) | 77 (14.6%) | 65 (25.6%) | 14 (35.9%) | <0.001 |

HBR = high bleeding risk; MACLE = major adverse cardiovascular and limb events.


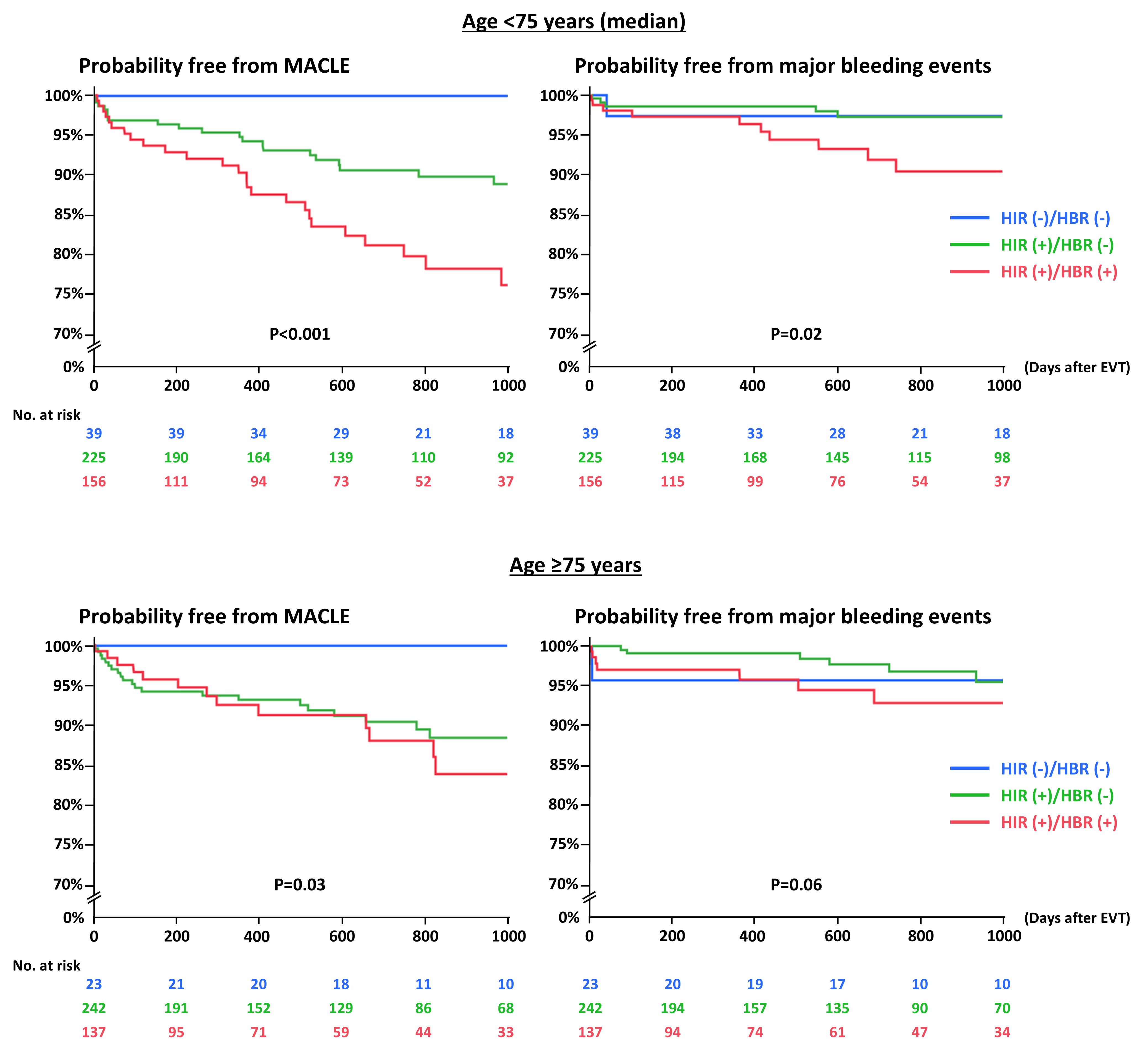


**Supplemental Figure 1. Stratified analysis of probability free from MACLE and major bleeding events by age**

EVT = endovascular treatment; MACLE = major adverse cardiovascular and limb events; HBR = high bleeding risk; HIR = high ischemic risk.


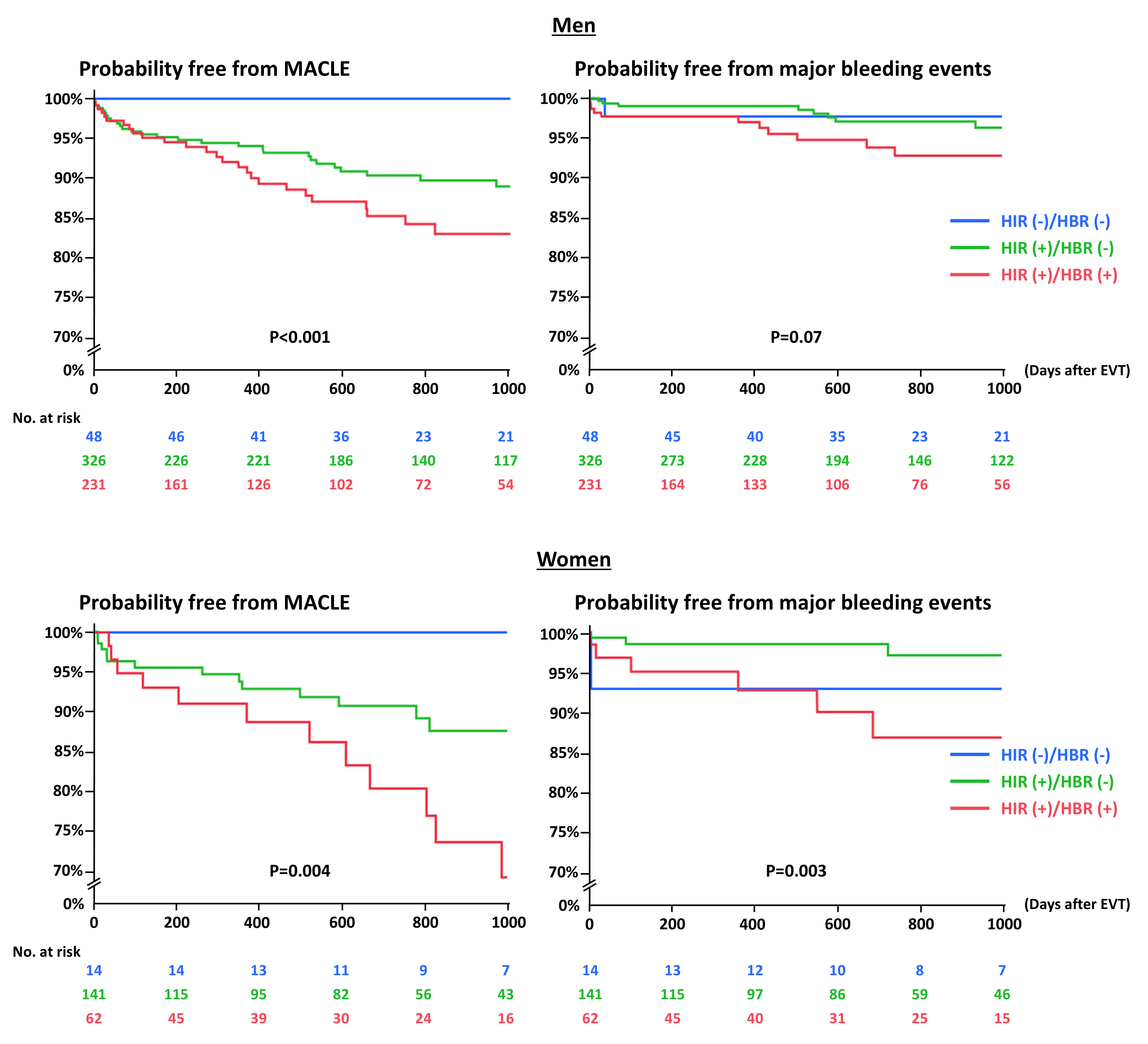


**Supplemental Figure 2. Stratified analysis of probability free from MACLE and major bleeding events by sex**

EVT = endovascular treatment; MACLE = major adverse cardiovascular and limb events; HBR = high bleeding risk; HIR = high ischemic risk.


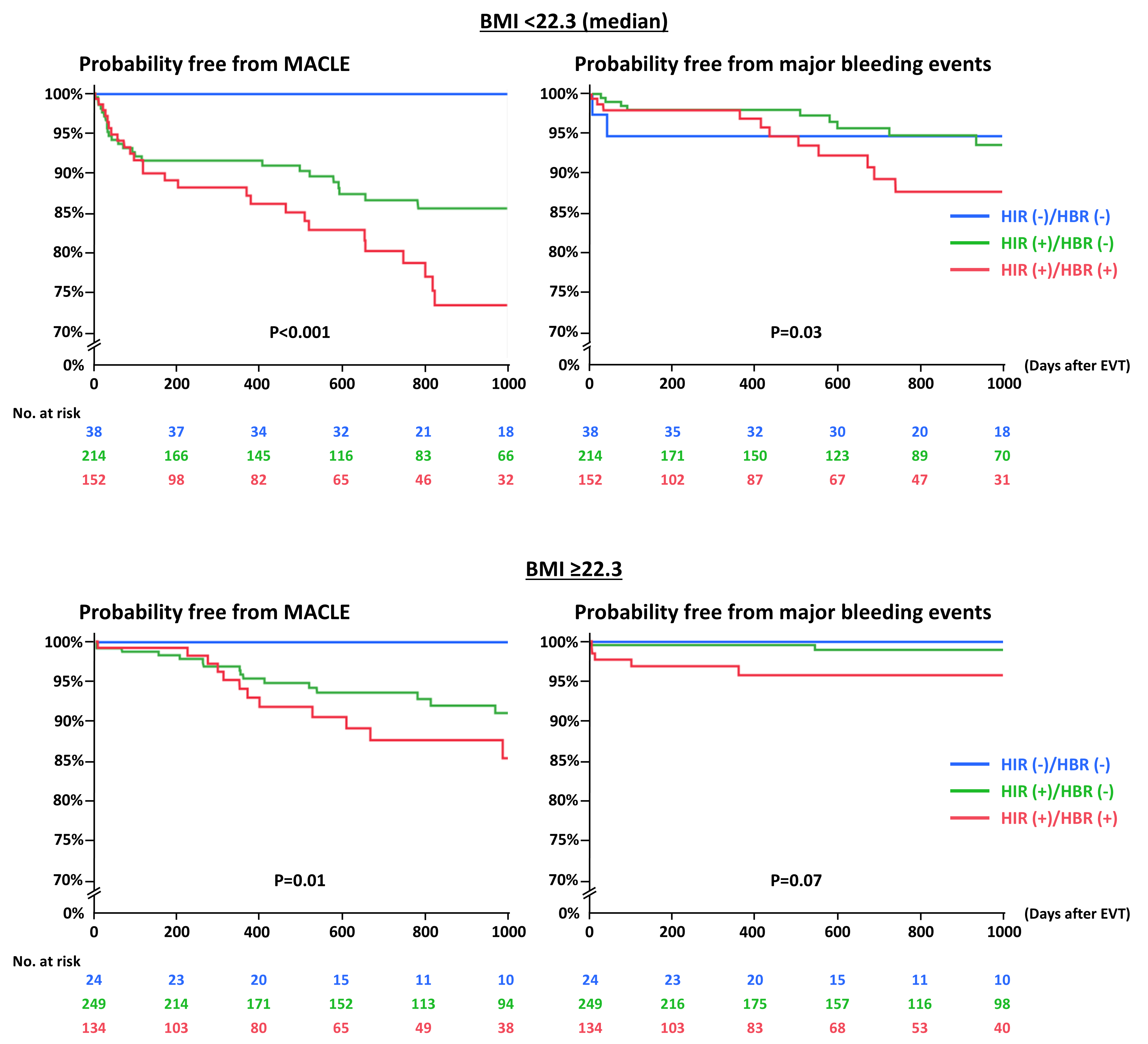


**Supplemental Figure 3. Stratified analysis of probability free from MACLE and major bleeding events by BMI**

BMI = body mass index; EVT = endovascular treatment; MACLE = major adverse cardiovascular and limb events; HBR = high bleeding risk; HIR = high ischemic risk.
